# Supplementary material for: Associations between Serum Saturated Fatty Acids Content and Mortality in Dialysis Patients
Source: J Clin Med. 2022 Aug 28;11(17):5051. doi: 10.3390/jcm11175051 (PMC9457217; doi:10.3390/jcm11175051)
Supplement: Supplementary file 1 [file jcm-11-05051-s001.zip › jcm-1833974-supplementary.pdf]

Journal of Clinical Medicine

**Table S1.** Baseline characteristics of the studied patients stratified according to the use of HMG-CoA reductase inhibitors (statins).

| | statin (n=29) | no statin (n=25) | p-value |
|---|---|---|---|
| **Age (years)** | 59.1 ± 11.4 | 53.8 ± 12.8 | 0.11 |
| **CRP (mg/l)** | 5.8 (1.4-12.3) | 3.6 (1.8-7.8) | 0.55 |
| **Albumin (g/l)** | 31.8 ± 4.1 | 32.4 ± 4.1 | 0.59 |
| **TC (mg/dl)** | 173 (140-208) | 182 (156-249) | 0.21 |
| **HDL (mg/dl)** | 39 (37-46) | 36 (31-44) | 0.08 |
| **LDL (mg/dl)** | 100 (73-130) | 115 (93-182) | 0.06 |
| **TG (mg/dl)** | 168 (78-208) | 161 (113-285) | 0.32 |
| **SFA (%)** | 34.1 ± 2.3 | 33.3 ± 2.8 | 0.13 |

CRP – high sensitivity C-reactive protein, DM – diabetes mellitus, CVD –cardio-vascular disease, TC – total cholesterol, HDL – high density lipoprotein cholesterol, LDL – low density lipoprotein cholesterol, TG – triglycerides, SFA saturated fatty acids.

**Table S2.** Baseline characteristics of the studied patients stratified according to their dialysis treatment.

| | PD (n=27) | HD (n=27) | p-value |
|---|---|---|---|
| **Age (years)** | 54.0 ± 11.6 | 59.7 ± 13.2 | 0.09 |
| **CRP (mg/l)** | 3.4 (1.4-7.8) | 6.8 (1.6-12.3) | 0.22 |
| **Albumin (g/l)** | 31.9 ± 4.7 | 32.4 ± 3.4 | 0.60 |
| **TC (mg/dl)** | 202 (167-245) | 177 (146-203) | 0.07 |
| **HDL (mg/dl)** | 38 (31-42) | 37 (35-49) | 0.40 |
| **LDL (mg/dl)** | 132 (93-167) | 106 (73-129) | 0.07 |
| **TG (mg/dl)** | 178 (127-228) | 149 (74-230) | 0.18 |
| **SFA (%)** | 34.0 ± 2.1 | 33.4 ± 3.0 | 0.22 |

PD – peritoneal dialysis, HD – hemodialysis, CRP – high sensitivity C-reactive protein, DM – diabetes mellitus, CVD – cardio-vascular disease, TC – total cholesterol, HDL – high density lipoproteincholesterol, LDL – low density lipoprotein cholesterol, TG – triglycerides, SFA saturated fatty acids.
